# Reflecting on professional identity in undergraduate medical education: implementation of a novel longitudinal course

**DOI:** 10.1007/s40037-021-00649-w

**Published:** 2021-03-09

**Authors:** Valérie Désilets, Ann Graillon, Kathleen Ouellet, Marianne Xhignesse, Christina St-Onge

**Affiliations:** 1 Department of Pediatrics, Faculty of Medicine and Health Sciences, Université de Sherbrooke, 3001, 12th Avenue North, Sherbrooke, Québec J1H5N4 Canada; 2 Centre for Health Sciences Education, Faculty of Medicine and Health Sciences, Université de Sherbrooke, Sherbrooke, Québec Canada; 3 Department of Family and Emergency Medicine, Faculty of Medicine and Health Sciences, Université de Sherbrooke, Sherbrooke, Québec Canada; 4 Department of Medicine, Faculty of Medicine and Health Sciences, Université de Sherbrooke, Sherbrooke, Québec Canada

**Keywords:** Reflection, Professional identity, Undergraduate medical education, Longitudinal course

## Abstract

**Background:**

Today’s healthcare professionals face numerous challenges. Improving reflection skills has the potential to contribute to the better management of complex patients and healthcare systems, as well as to improve professional practice. However, the question of how reflection skills can inform professional identity development at the undergraduate medical education level remains unanswered.

**Approach:**

The authors developed and implemented a 4-year course that aims to engage students in a reflective process to increase their awareness of their professional identity development. The course is structured around three types of pedagogical activities: workshops, reflections deposited in an electronic portfolio, and individual discussions with mentors.

**Evaluation:**

Sixty-four 1st year students (33%) and 17 mentors (50%) from the 2017–2018 cohort completed evaluation questionnaires. For the 2018–2019 academic year, 73 1st year students (34%) and 27 2nd year students (14%), as well as 20 1st year (59%) and 19 2nd year mentors (57%) replied. Students and mentors considered that the pedagogical activities contributed to the development of students’ professional identity through the acquisition of reflection skills, but some elements were perceived as challenging, notably, completing the portfolio, finding a subject to reflect about and the timing of the proposed activities.

**Reflection:**

An important preoccupation when wanting to foster the development of professional identity through the acquisition of reflection skills is the authenticity of students’ reflection. We tried to favor authentic reflection, by having a mentee-mentor pair throughout the entire 4‑year course. A rigorous evaluation process helped us identify and promptly correct issues as they surfaced.

**Supplementary Information:**

The online version of this article (10.1007/s40037-021-00649-w) contains supplementary material, which is available to authorized users.

## Background and need for innovation

Today’s healthcare professionals face numerous challenges including a significant increase in knowledge, complex health problems, and the evolution of healthcare systems. Optimizing reflection skills in healthcare professionals could contribute to the better management of complex patients and healthcare systems [1], improve professional practice, and also limit diagnostic errors [2]. Thus, reflection on one’s professional practice is increasingly seen as an essential skill, as it is thought to prepare students for lifelong learning [3].

High-quality guidance and feedback to help students understand the goal of reflection [4] has been identified as important to foster the development of reflection skills at the undergraduate medical education (UGME) level. Other success factors include the presence of a safe environment, mentorship, peer support, and time to reflect [1]. Strategies to facilitate this development of the skills of reflection include supporting self-insight, creating a safe environment, and encouraging students to take responsibility for their own development [5].

## Goal of innovation

Based on the aforementioned considerations, we implemented a novel course at the UGME level to engage students in a reflective process to increase their awareness about their professional identity development.

## Steps taken for development and implementation of innovation

We developed a four-year longitudinal course within our UGME program, which allows for early clinical exposure. The decision to adopt a longitudinal approach to the development of professional identity through the acquisition of reflection skills was rooted in the theory of professionalization informing our curriculum renewal [6]. According to this theory, students should not only acquire knowledge, but have early exposure to clinical settings and reflect on their experiences. The course, comprised of three types of pedagogical activities, is informed by Kolb’s [7] experiential learning framework (see Fig. 1 in Electronic Supplementary Material [ESM]). This framework allows for investment of acquired learnings. The learning activities can be individual or collective in nature, but the learning that students acquire from these is individual. In our course, students engage in a reflexive process through activities thought to trigger their development in a safe, small group environment, based on topics recently tackled in other settings embedded within the UGME curriculum or experienced in their personal lives. Through these pedagogical activities, we aim to provide students with an opportunity to develop their professional identity through the acquisition of reflection skills so that they can subsequently mobilize them in their professional practice.

In the context of this course, reflection is defined—building on Sandars’ work [8]—as “a metacognitive process including connecting with feelings that occurs before, during and after situations with a purpose of developing greater awareness and understanding of self, other, and situation, so that future encounters with the situation including ways of being, relating, and doing are informed from previous encounters” [9].

Students’ reflection during the course is on their professional identity, as conceptualized by Cruess and Cruess [10], which translates as a “representation of self, achieved in stages over time during which the characteristics, values and norms of the medical profession are internalized, resulting in individual thinking, acting and feeling like a physician” [10, p. 1447]. Socialization is a key contributor to professional identity formation [11]. We conceptualized and implemented a reflection process on the development of professional identity as being comprised of four elements: relationship with self (identification with a role), situations (clinical, learning), profession (appropriation of values and norms), and society.

The course, illustrated in Fig. 1 (in ESM), is structured around three types of pedagogical activities occurring within a 1-week span, three or five times per year. These activities are a) workshops on a theme, b) structured reflection exercises deposited in an electronic portfolio, and c) discussions with a mentor. Each workshop lasted 90 min and individual meetings with a mentor lasted 30 min.

### Workshops

Group discussions are composed of six students and one mentor for the duration of the UGME program. Topics were selected for their potential to trigger reflection on professional identity and correspond to recently viewed content or clinical experiences. As recommended by Boud and Walker [12], we also paid great attention to context when choosing the workshop themes. Therefore, topics were adapted to students’ developmental needs and role transitions (medical student, clerk, future resident). Themes, per year, are listed in Fig. 1 (in ESM). Workshops are held five times the 1st year and three times the following years.

### Reflections deposited in an electronic portfolio

Students document their experiential learning (i.e., their experiences that brought on learning) using an electronic portfolio. To better capture the full experience, including students’ feelings, reflections can take different forms (e.g., texts, videos, images). Students are invited to reflect in preparation for their individual discussions with their mentor and, afterwards, identify next steps for their development.

### Individual discussions with a mentor

These discussions consist of one-on-one sessions throughout the four-year program to support the gradual development of their professional identity through the acquisition of reflection skills. Students are invited to express their emotions and reflections in these meetings, and to document them in their electronic portfolio. Meetings with the mentor happen a few days after the small group discussions. By depositing their reflections in their portfolio, students can structure them, prepare for the mentor encounter, and keep track of their thoughts, actions, and feelings.

### Student assessment

Students are given a Pass or Fail grade for the course. This grade does not consider the content of the students’ reflections, but rather their commitment to the process of developing their professional identity through the acquisition of reflection skills. The mentors provide verbal and written feedback to the students throughout the year and identify strengths, difficulties, and, where applicable, any concerns, via a narrative assessment [13]. More specifically, they comment on the student’s commitment to reflection (e.g., preparedness, openness to different points of view) and their use of a reflective process informed by Kolb’s framework (e.g., the student is able to describe a situation which raises a question and take a critical distance to discuss it).

## Outcomes of the innovation

A standard program evaluation questionnaire was sent to all 1st year students (196) and mentors (34) at the end of 2017–2018 and all 1st year students (215) and 2nd year students (189) and all mentors (67) at the end of 2018–2019. It mostly consisted of Likert-type questions (4-point scale from “strongly agree” to “strongly disagree”, 17 questions for students, 12 questions for mentors) structured around the following themes: course plan, preparation, mentors’ contribution, portfolio, and general appreciation. Both questionnaires included five open-ended questions. For this paper, we report on those questions that specifically relate to the innovation discussed. Data were provided to us by the UGME program following ethical approval by our local Institutional Review Board (on 14 August 2017 by the Comité d’éthique de la recherche (CÉR)—Éducation et sciences sociales, Université de Sherbrooke, ID number: 2017-1485/St-Onge).

Descriptive statistical analyses were done on the quantitative data. The qualitative data were the subject of a thematic analysis. One team member (KO) categorized comments around five themes: 1) themes outlined in the course, 2) portfolio, 3) relationship with mentor, 4) development of reflection skills, and 5) implementation. This coding structure was revised by a team member (CSO) and presented to all co-authors, with supporting excerpts.

For 2017–2018, 64 students (33%) and 17 mentors (50%) completed the questionnaire. For 2018–2019, 73 1st year students (34%) and 27 2nd year students (14%) completed the questionnaire as well as 20 1st year mentors (59%) and 19 2nd year mentors (57%). Tab. 1 presents mean scores for both students’ and mentors’ evaluations. Students and mentors both appreciated the themes discussed and considered the pedagogical activities as contributing to the development of their professional identity through the acquisition of reflection skills. Students appreciated the relationship with their mentor, even more so in their second year. Neither students nor mentors appreciated the portfolio in the first two years.

**Table 1:** Mean (M) and standard deviation (SD) per evaluation item for both mentor and students

Statements	Students			Mentors		
	2017–2018	2018–2019		2017–2018	2018–2019	
	1st year (*n* = 64)	1st year (*n* = 73)	2nd year (*n* = 27)	1st year (*n* = 17)	1st year (*n* = 20)	2nd year (*n* = 19)
	M (SD)	M (SD)	M (SD)	M (SD)	M (SD)	M (SD)
*Themes*						
The themes discussed during the workshops are good starting points for my [students’] reflection about the development of my [their] professional identity	3.32 (0.62)	3.18 (0.63)	3.04 (0.65)	3.76 (0.44)	3.95 (0.22)	3.63 (0.60)
*Portfolio*						
Once completed, the portfolio helps me reflect on the development of my practice and my professional identity	2.13 (0.93)	2.66 (0.84)	2.37 (0.88)	N/A	N/A	N/A
For most of the students, the portfolio helps support and record reflections on their practice and professional identity	N/A	N/A	N/A	2.82 (1.01)	3.05 (0.69)	2.68 (0.67)
*Relationship with the mentor*						
The mentor fosters a climate of openness that is conducive to learning	3.42 (0.79)	3.48 (0.80)	3.74 (0.45)	N/A	N/A	N/A
The meetings with my mentor help me improve my reflection skills	3.47 (0.76)	3.37 (0.86)	3.70 (0.54)	N/A	N/A	N/A
My relationship with my mentor is satisfactory	3.41 (0.79)	3.40 (0.97)	3.74 (0.53)	N/A	N/A	N/A
*Development of reflection skills (General appreciation)*						
I feel comfortable sharing my reflections during the course; I don’t hold anything back	3.16 (0.82)	3.08 (0.94)	3.26 (0.76)	N/A	N/A	N/A
The evaluation of the course is a source of feedback that contributes to my reflection on the development of my practice and my professional identity	2.97 (0.85)	3.03 (0.85)	2.81 (0.74)	N/A	N/A	N/A
Overall, the students embrace reflective practice	N/A	N/A	N/A	2.94 (0.83)	3.10 (0.64)	3.04 (0.52)
The proposed activities generally encourage the students to engage in their reflective practice	N/A	N/A	N/A	3.35 (0.70)	N/A	N/A

We structured our qualitative data around the five themes previously mentioned.

### Themes

The themes proposed as a starting point for reflection are considered relevant for the development of a reflective practice.

*“The workshop themes are well chosen and allow a good reflection.” *(student)

*“All the themes are really relevant.” *(mentor)

### Portfolio

The students’ comments indicate that they did not find the portfolio to be very user-friendly. Mentors’ comments are consistent with this view. They note that the portfolio tool is difficult to use.

*“The portfolio stresses me out because it’s so hard to use!” *(student)

*“The tasks to be done in the portfolio could be clearer.” *(mentor)

### Relationship with mentor

Students’ comments emphasize the importance of the relationship with their mentors. Mentors also consider their relationship with students to be central.

*“For me, having a mentor has been very reassuring, especially since we change tutors regularly. The mentor is someone I can rely on.” *(student)

*“The positive impact is in the relationship.”* (mentor)

### Development of reflection skills

In terms of developing reflection skills, students recognize the relevance of the proposed activities and emphasize the importance of setting aside time to develop these skills. However, several students report that if they haven’t encountered a specific problem on which they consider they need to reflect, they will make up a situation. This practice is worrisome in that it compromises the authenticity of their reflections.

*If I ’m being very honest, this course makes me feel like I have to invent problems, so I have something to talk about with my mentor. *(student)

Some of the mentors also recognized having difficulty helping students deepen their reflections when providing written feedback.

### Implementation

We identified some barriers to the implementation of the course. For example, several students report having to do too much reflection during the course (too many meetings with mentors) and throughout the entire program (in other courses). Some mentors agreed with students:

*Workshops and meetings with mentors become repetitive. We do not know what to discuss. *(mentor)

Many students also think the timing of activities (during integration week right before exams) is not ideal.

*During integration week, I’d rather spend my time studying and preparing my exams than reflecting, so I think the timing of the workshops could be better. *(student)

The mentors agree that some course activities should be moved to a different time:

*Scheduling the workshops right before their exams hinders the reflection process. *(mentor)

## Critical reflection

An increasing number of programs are introducing reflective activities into their curricula. We adopted the following recommendations when creating our course that aims to encourage the development of professional identity through the acquisition of reflection skills: provide feedback and a safe environment for students [1, 4, 14], embrace diversity in reflections [3], and make sure students understand the purpose of reflective practice [4]. In addition, we built in a longitudinal aspect to our course to favor the development of reflective skills over time such as to better inform professional identity development. Overall, our results are consistent with success factors known to foster the development of reflection skills such as providing a safe environment, mentorship, peer support, and time to reflect.

A potential pitfall in the effort to foster the development of reflective skills, whether related to the development of professional identity or not, is to create “reflective zombies” as suggested by De la Croix and Veen [3]. We tried to favor authentic reflection and the development of lifelong reflective skills by having a mentee-mentor pair throughout the entire 4‑year course. We encouraged students and mentors to consider positive experiences and key transition points (e.g. the start of clerkship) in their reflections. Like Mann et al. and Chambers et al. [1, 4], we also believe that helping students see the potential benefits of reflective practice may help them truly engage in their own development.

The implementation of this course has gone through a rigorous evaluation process and the results were presented to an advisory committee. We were able to identify and promptly correct issues as they surfaced, for example, we made changes to the assessment form (number of cells to fill out), and to the guidelines given to mentors to complete the form. The electronic portfolio was also greatly simplified. Further faculty development seems necessary to help mentors welcome each and every student reflection and help them go further in the process. Finding the right moment in a curriculum for a reflective activity remains a challenge; thus, we continue to explore potential changes in the timing of activities to avoid any potential duplication. As recommended by Sandars [8], we are aware of the importance of seeing reflection as a process connected with the content of our curriculum. A preoccupation that remains is the intended/unintended and positive/negative consequences of the assessment embedded in the course. We are currently conducting a qualitative descriptive study where students, interviewed individually, are asked to discuss their perceptions of the assessment component of the course.

The outcomes reported in this paper are based on evaluation questionnaires with students and mentors. We recognize that response rates for 2nd year students are low but still believe these data are informative in the context of quality monitoring. We plan to continue to meet annually with an advisory committee to continue to explore potential areas for improvement.

## Supplementary Information

Figure 1. Diagrammatical overview of the 4-year longitudinal course
