# Supplementary figures and images for: Reflecting on professional identity in undergraduate medical education: implementation of a novel longitudinal course
Source: Perspect Med Educ. 2021 Mar 9;11(4):232–6. doi: 10.1007/s40037-021-00649-w (PMC9391548; doi:10.1007/s40037-021-00649-w)

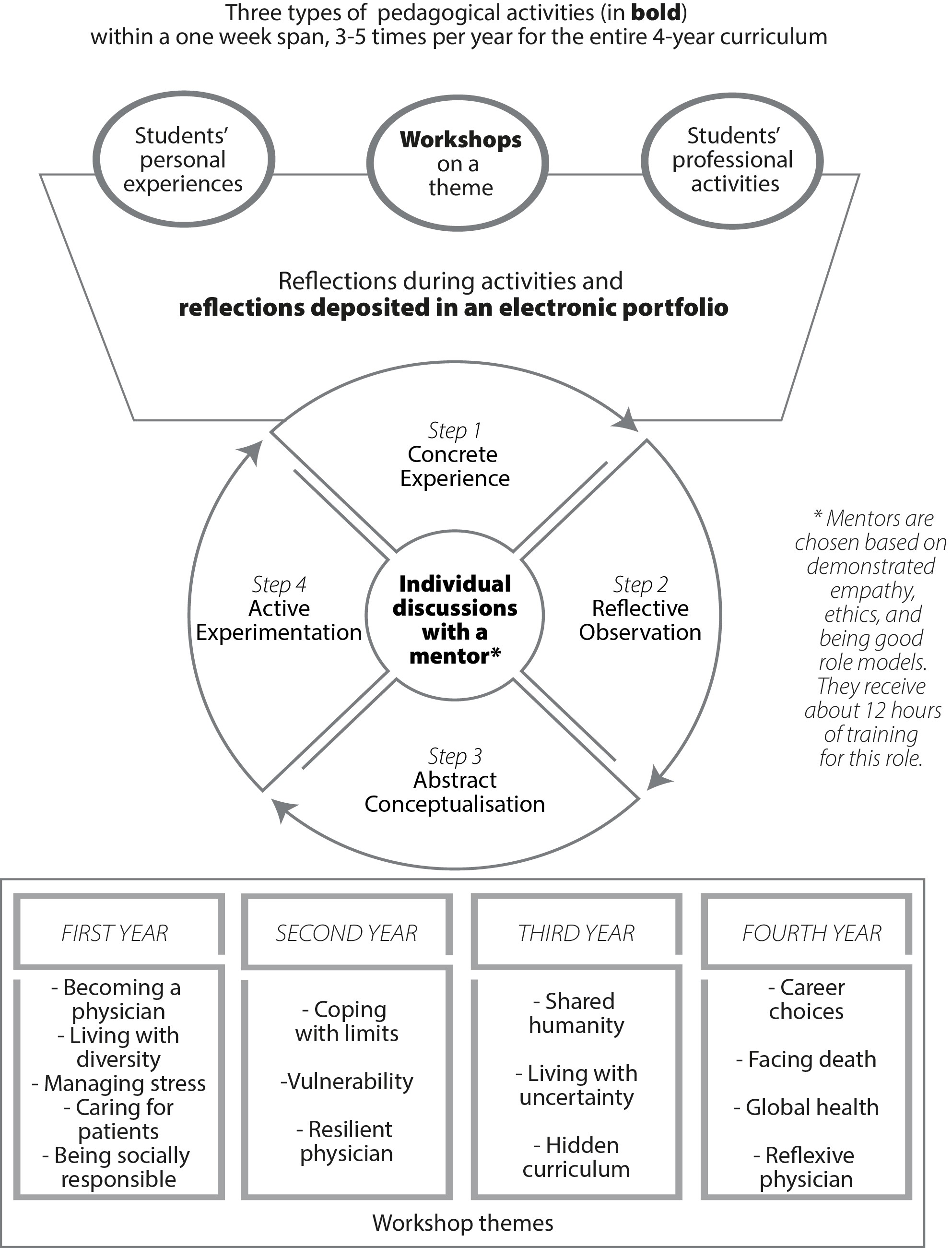

Supplement: Supplementary file 1 — Figure 1. Diagrammatical overview of the 4-year longitudinal course [file 40037_2021_649_MOESM1_ESM.jpg]
